# An analog of Friedel oscillations in nanoconfined water

**DOI:** 10.1093/nsr/nwab214

**Published:** 2021-11-29

**Authors:** Minmin Xue, Zhili Hu, Hu Qiu, Chun Shen, Wanlin Guo, Zhuhua Zhang

**Affiliations:** Key Laboratory for Intelligent Nano Materials and Devices of Ministry of Education, State Key Laboratory of Mechanics and Control of Mechanical Structures, and Institute for Frontier Science, Nanjing University of Aeronautics and Astronautics, Nanjing 210016, China; Key Laboratory for Intelligent Nano Materials and Devices of Ministry of Education, State Key Laboratory of Mechanics and Control of Mechanical Structures, and Institute for Frontier Science, Nanjing University of Aeronautics and Astronautics, Nanjing 210016, China; Key Laboratory for Intelligent Nano Materials and Devices of Ministry of Education, State Key Laboratory of Mechanics and Control of Mechanical Structures, and Institute for Frontier Science, Nanjing University of Aeronautics and Astronautics, Nanjing 210016, China; Key Laboratory for Intelligent Nano Materials and Devices of Ministry of Education, State Key Laboratory of Mechanics and Control of Mechanical Structures, and Institute for Frontier Science, Nanjing University of Aeronautics and Astronautics, Nanjing 210016, China; Key Laboratory for Intelligent Nano Materials and Devices of Ministry of Education, State Key Laboratory of Mechanics and Control of Mechanical Structures, and Institute for Frontier Science, Nanjing University of Aeronautics and Astronautics, Nanjing 210016, China; Key Laboratory for Intelligent Nano Materials and Devices of Ministry of Education, State Key Laboratory of Mechanics and Control of Mechanical Structures, and Institute for Frontier Science, Nanjing University of Aeronautics and Astronautics, Nanjing 210016, China

**Keywords:** ion sieving, Friedel oscillations, confined water, water transport

## Abstract

Water confined in nanometer-scale crevices and cavities underpins a wide range of fundamental processes, such as capillary flow, ion transport and protein folding. However, how water responds within these confined spaces, with prevalent inhomogeneity built in or caused by impurities, is not well understood. Here, we show theoretically that water confined in one-dimensional nanochannels with localized perturbation exhibits pronounced density oscillations. The oscillations occur vividly like the Friedel oscillations in electron density resulting from defects in metals. A model analysis reveals that the density oscillations result from the perturbation-induced molecular scattering that is augmented by the confinement-enhanced correlation of water dipoles. This renders the oscillations a general behavior independent of the channel geometries and specific forms of the perturbation. Under confinements comparable to biological ion channels, such oscillations can strikingly extend over 10 nm, resulting in non-trivial effects at large distances that, for example, repel all ions from the channels with their long-range force. These results deepen the understanding of biological functions and inspire new applications in a variety of domains, such as ionic sensing and seawater desalination.

## Teaser

Water confined in nanochannels with localized perturbations exhibits Friedel-like density oscillations that can repel ions from the channels with their long-range force, with implication for applications in ionic sensing and seawater desalination.

## INTRODUCTION

The field of condensed matter physics has long sought to understand how (quasi-)particles in liquid(-like) systems interact when subjected to a local disturbance. A notable example of such an interaction is the spatially decaying, standing-wave-like modulations in electron density of a metal by a localized perturbation (e.g. defect) in the system. These phenomena in a Fermi liquid are known as Friedel oscillations [1]. Similar phenomena can become more pronounced in one-dimensional (1D) physical systems [2], where the Fermion behaviors change from a Fermi liquid to a Luttinger liquid due to non-negligible interactions between the Fermions [3]. Successful descriptions of Friedel oscillations have not only led to the decoding of hidden physics in low-dimensional metallic systems [4,5], but have also led to a wide range of practical applications in, for example, photonics and electronics [6–8].

A question arises, then, as to whether an analog of Friedel oscillations exists in one-dimensionally confined water. This question is relevant since water confined in nanometer-scale pores and cavities is ubiquitous in natural and synthetic systems, such as plants, soils, skin tissues, porous materials and proteins [9–12]. Spatial confinement has been shown to cause a series of oddities in the structures and properties of water [13–22]. One of the most remarkable features of confined water lies in its ordered molecular structures, which give rise to the enhanced collective movement of water molecules inside the nanochannels [23–30], much like interacting electrons in 1D nanoconductors [3,31,32]. This microscopic similarity suggests that quantum-like phenomena may arise in the confined water in the presence of a localized perturbation.

Unfortunately, our current understanding of confined water is largely based on nanoconfinements with smooth walls that do not reflect the practical complexity of most channels infused with water. More often than not, water in natural and artificial channels is subject to various perturbations from either intrinsic defects, bumps and pleats, or from the extrinsic contaminants on the walls. A recent study suggested that the heterogeneity of channel surface charges can considerably influence the slip length of nanoconfined water [33]. In this work, we report computer simulation evidence of a synergy between 1D confinement and localized perturbation triggering pronounced density oscillations in water with a distance-dependent decay, exhibiting profiles closely resembling Friedel oscillations. Then, instead of fighting perturbation in nanoscale passages, we embrace it to remotely control the ionic transport by taking advantage of density oscillations extending up to 10 nm. These results are expected to be generic to other polar liquids, potentially establishing a new, fundamental characteristic of conventional liquids.

## RESULTS

### Atomistic simulations

**Figure 1.**
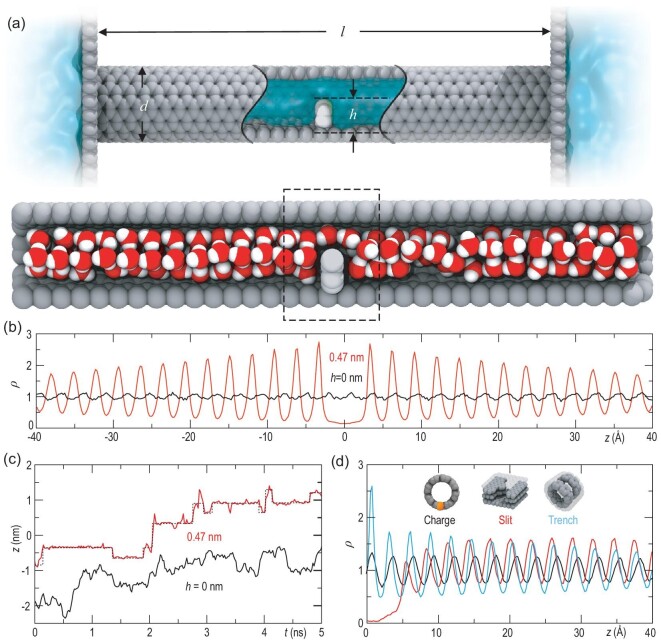
Simulated density oscillations of confined water in locally perturbed nanochannels. (a) Top panel: the model system consisting of a CNT channel (gray) and water reservoir (cyan) separated by graphene sheets at both ends of the channel. The channel has a length of *l*, a diameter of *d* and a baffle with a height of *h* (light gray) at its middle. Bottom panel: water in the channel, with oxygen and hydrogen atoms colored in red and white, respectively. (b) Density of water oxygens in a perfect CNT and in a (8,8) Δ-CNT with *h* = 0.47 nm. (c) Evolution of axial coordinates of a randomly chosen oxygen atom in the (8,8) CNTs with *h* = 0 (black) and 0.47 nm (red). The dotted line is to guide the eye for the stepped variation of the oxygen coordinate. (d) Density oscillations in channels with different forms of the local perturbations. Cross sections of these locally perturbed channels are shown in insets, including a charge (colored orange), a circular trench inside a double-walled CNT and a slit nanopore with a local protrusion.

We study the effect of perturbations on the structure of water confined in nanochannels by carrying out full-atom molecular dynamic (MD) simulations. We first employ carbon nanotubes (CNTs) to stand in for the nanochannels, with a fixed baffle in the middle of the CNT for inducing the perturbation (Fig. 1a). Other nanochannels with distinct forms of perturbations will be considered later (see Fig. 1d). Such locally perturbed CNTs are denoted as Δ-CNTs, and have a diameter of *d*, a length of *l* and a baffle height of *h*. The Δ-CNT is connected to water reservoirs at both ends. The structures of confined water are characterized mainly by calculating the time-averaged density of water oxygens, *ρ*(*z*), where *z* is the coordinate along the channel axis. For ease of comparison, *ρ*(*z*) is normalized by the mean density of oxygens in the occupiable space within the Δ-CNT. More details on the methods and simulations are provided in the Methods section.

Our simulations reproduce previous results on the water structure confined in a perfect (8,8) CNT [34], in which *ρ*(*z*) exhibits a slight fluctuation due to the lattice potential of the CNT wall (Fig. 1b) at a temperature *T* = 300 K. *ρ*(*z*) is qualitatively changed by a baffle with *h* = 0.47 nm in the CNT, showing a pronounced oscillation and a noticeable decay as it moves away from the baffle. Such a profile of *ρ*(*z*) is striking in its resemblance to the Friedel oscillations in Fermi liquids, where a defect induces a localized perturbation that scatters electrons and results in a rippling pattern around the defect (Fig. S1 for comparison). The density oscillation is further confirmed by tracing the *z* coordinate of a randomly picked water molecule in the (8,8) Δ-CNT; the thermal fluctuation makes the molecule translocate in a stepped manner (Fig. 1c), like electronic hopping between distinct atomic sites. This feature stands in contrast to the continuous variation of the corresponding *z* coordinate in a perfect (8,8) CNT.

Decreasing the height *h* weakens the oscillation intensity, but the decay rate remains essentially the same for the Δ-CNTs with a given *d*; whereas increasing the CNT–water interaction enhances the oscillation intensity (Fig. S2). Note that the oscillation remains in place when the structure of confined water becomes highly disordered by increasing *T* to 325 K (Fig. S3a), at which the calculated radial distribution function and structural factor of oxygen support a liquid water structure (Fig. S3). The oscillation will not disappear until the confined water approaches a nearly discrete gaseous state at a temperature of up to 375 K (Fig. S3a, top inset). Therefore, the density oscillations are an intrinsic behavior to liquid water, with no apparent connection with ice.

Further simulations verify the oscillations in graphene slit nanopores that have a square-shaped cross section if there is a protruded benzene ring or a baffle inside the nanopores (Fig. 1d and Fig. S4). More importantly, the perturbation source is not limited to protrusions but applied to more general forms, such as a charged impurity on the CNT wall, a locally necked CNT and a circular trench inside a double-walled CNT (Fig. 1d and Fig. S4). All these density oscillations can spread throughout the confined space with no end in sight. It is interesting that all the oscillations in different cases have an almost uniform wavelength of *λ* = 2.9 Å, namely the average distance between the oxygens of two neighboring molecules. This result indicates that the wavelength is determined by the characteristic size of molecules, echoing the wavelengths of Friedel oscillations decided by electronic sizes—the Fermi wavelengths.

The oscillations from two or more perturbations can be superimposed. This results in an enhanced oscillation intensity of *ρ* over that from a single perturbation, as shown in Fig. S5—which shows the case of charges residing on a physical baffle. Thus, we expect that multiple perturbations coexisting in nanochannels would mutually produce much more pronounced oscillations inside the nanochannels. Interestingly, similarly enhanced oscillations appear to have been spotted in a single-file water chain confined in aquaporin proteins in a previous experiment [35,36], likely due to coexisted local charges and spatial narrowing in the proteins.

### Model analysis

**Figure 2.**
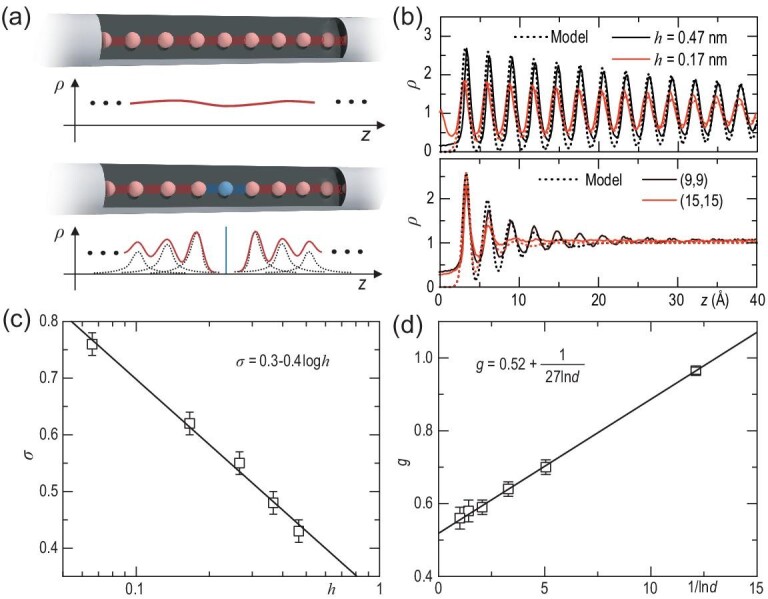
An analytical model for the density oscillations. (a) Schematic of (top) 1D and (bottom) fractionally confined systems and the corresponding time-averaged density. The perturbed source is colored blue and the interactions between molecules are represented by springs. The red curve shows the sum of density distributions *ρ_i_* (dashed lines). (b) Comparison between the analytical solutions and simulation results. (c) Logarithm relationship between *σ* and *h*. Error bars are estimated from *ρ*_±1_ of the best fitted curves. (d) Linear relationship between *g* and the reciprocal of natural logarithm of *d*. Error bars for *g* are estimated from the best fitted *ρ*.

We derive a physical model to understand the density oscillations of confined water. This model is established by regarding water molecules as particles, which, when trapped in a potential well, obey a probability density distribution, *ρ*, around a position at the well's bottom. Under 1D confinement, water molecules are restricted to translocating along *z*, forming axial layer-by-layer—or even molecule-by-molecule—structuring [28] (Fig. 2a, top). Then, each molecule strongly correlates to its neighbors by a relatively rigid network of hydrogen bonds. Yet, time-averaged features of the confined water degenerate almost into a flat line due to the axial translocation of molecules with time, as is illustrated by *ρ* in Fig. 2a.

A local impurity inside the channels perturbs the molecular translocation by introducing a well (Fig. 2a, bottom). The water molecules very near to the impurity are trapped and form a distinct peak in *ρ*. Owing to enhanced molecular correlation, such a degree of immobilization of these molecules directs their neighbors one-by-one away from the impurity. As a result, water molecules at a given *z* will show a characteristic density distribution, as illustrated by the dashed lines in Fig. 2a, bottom. In a way, the described system exhibits the characteristics of both 1D and 0D confined water and, hence, can be termed a fractional confined system. If we approximate the potential well for each water molecule to be a quadratic function, we can derive *ρ_i_* of *i*th trapped molecule by solving the Fokker-Planck equation, which reads as a normal distribution

(1) \begin{equation*} {\rho _i}\left( z \right) = \frac{1}{{{\sigma _i}\sqrt {2\pi } }}{e^{\frac{{ - {{\left( {z - {\mu _i}} \right)}^2}}}{{2{\sigma _i\!\!\!}^2}}}}, \end{equation*}

where *μ_i_* is the axial position of the bottom of the *i*th potential well and *σ_i_* is the scale parameter of the Gaussian distribution. Note that *ρ*_±1_ and *σ*_±1_ correspond to those molecules adjacent to the impurity on both sides. We can express *ρ* for all the confined water as a sum of a series of *ρ_i_* at different *z_i_*

(2) \begin{equation*} \rho \left( z \right) = \sum\limits_{i = - N,i \ne 0}^N {\frac{1}{{{\sigma _i}\sqrt {2\pi } }}{e^{\frac{{ - {{\left( {z - {\mu _i}} \right)}^2}}}{{2{\sigma _i\!\!\!}^2}}}}} . \end{equation*}

Prior to solving *ρ*, it is worth stressing two important factors: the perturbation strength and the molecular correlation. Directly parameterizing the two factors is possible, yet challenging. To circumvent this issue, we introduce two parameters, *σ* = *σ*_±1_ and *g* = *σ*_|_*_i|_*/*σ_|i|_*_+1_, to characterize the two factors, respectively. Then, we rewrite Equation (2) as

(3) \begin{equation*} \rho \left( z \right) = \sum\limits_{i = - N,i \ne 0}^N {\frac{\lambda }{{\sigma /{g^{\left| i \right| - 1}}\sqrt {2\pi } }}{e^{\frac{{ - {{\left( {z - i\lambda } \right)}^2}}}{{2{{(\sigma /{g^{\left| i \right| - 1}})}^2}}}}}} , \end{equation*}

where *λ* = 2.9 Å. Thus, *ρ* (normalized over the mean density) in a locally perturbed nanochannel becomes an oscillating profile (Fig. 2a, bottom). Unlike electrons, the limited diffusivity of water molecules allows them only to exchange positions rather than to disperse over a wide space. Thus, *σ_i_* has an upper limit (*σ_i__ _*< 2, Fig. S6) to avoid an overly wide distribution of density.

With suitably chosen *σ* and *g*, *ρ*(z) derived from our model almost reproduces simulation results, as shown in Fig. 2b and Fig. S7. The major difference is slightly deeper valleys compared to those in the simulated profiles. This can be rationalized by the molecular exchange in simulations and an empty occupation at the perturbed site in the model. Starting from the perturbed site, the oscillation sustains for up to ∼8 nm in the (8,8) Δ-CNT, close to ∼10 nm as was estimated by simulations. Moreover, a smaller *σ* means a stronger perturbation, resulting in a sharper *ρ*_±1_ and a more remarkable oscillation, as reflected by simulations with different *h*. The analyzed density oscillations in the (6,6) Δ-CNT are more pronounced than those from simulations; the oscillations become a rough and prickly curve with a decay length of 3–4 nm (Fig. S8). The slight discrepancy between theory and simulations is caused by frequent breaking and retreating of the single-file water chain towards the tube ends. More rapidly decaying oscillations in larger Δ-CNTs, such as (9,9) and (15,15) CNTs, for a given *h*/*d* = 0.5 (Fig. 2b, bottom) are also reproduced by our model. The decay rate, *g*, is found to decrease with increasing *d*. Yet, the oscillations still extend for ∼2 nm in the (9,9) CNT (*d* = 1.22 nm), comparable to characteristic lengths of biological ion channels of similar sizes.

To gain deeper insight into the density oscillations, we fitted all the simulated profiles for Δ-CNTs using Equation 3. We explored the correspondence of fitting parameters, *σ* and *g*, to simulation parameters. Figure 2c shows that *σ* linearly decreases with increasing log(*h*), approximately in a function of *σ* = 0.3–0.4 log(*h*). Water–water correlation was investigated by looking into the hydrogen bond network in the confined condition. The donor-acceptor dipole orientations show conspicuous consistence along the *z* axis of the Δ-CNTs, although the average number and lifetime of hydrogen bonds show negligible difference between the two cases with and without density oscillations. Therefore, we describe the hydrogen bond network by calculating the dipole correlation that is described by an average cosine value of the angle *θ* formed between dipole vectors of two adjacent waters along the *z* direction, 〈cos*θ*〉. The calculated 〈cos*θ*〉 is a linear function of fitted *g*, expressed as *g* = 2 〈cos*θ*〉 (Fig. S9). Since 〈cos*θ*〉 across all systems involved in this work is a unary function of the tube diameter *d*, *g* can be further expressed as *g* = 0.52 + 1/27 ln(*d*). Note that *h* and *d* are non-dimensionalized values by the unit length of nm for simplicity. These results rationalize our model in that *σ* and *g* indeed characterize the localized perturbation and molecular correlation, respectively. Therefore, *ρ*(*z*) is determined by only two geometrical parameters, *d* and *h*. As such, *ρ*(*z*) of water in a given Δ-CNT can be obtained without any fitting parameters as

(4) \begin{equation*} \left\{ {\begin{array}{@{}*{1}{c}@{}}\displaystyle {\rho \left( z \right) = \sum\limits_{i = - N,i \ne 0}^N {\frac{\lambda }{{{\varphi _i}\sqrt {2\pi } }}{e^{\frac{{ - {{\left( {z - i\lambda } \right)}^2}}}{{2{\varphi _i\!\!\!}^2}}}},} }\\ \displaystyle{{\varphi _i} = \frac{{(0.3 - 0.4\log h)}}{{{{(0.52 + \frac{1}{{27\ln d}})}^{\left| i \right| - 1}}}}.} \end{array}} \right. \end{equation*}

### Hydrodynamic case and ion sieving

The density oscillations also exist in nanofluidic water moving under a pressure gradient through the perturbed channels (Fig. S10). The major difference is that the flow breaks the oscillation symmetry with respect to the baffle. Compared to the static case, the flowing water shows an enhanced oscillation on the inflow side but a weakened one on the outflow side. On the inflow side, the peaks of *ρ_i_* shift slightly toward the impurity due to the impact of water molecules attempting to flow in, resulting in a slightly shorter *λ*. The shorter *λ* caused by an external pressure is analogous to the change in Fermi wave vector *k*_F_ in 1D quantum systems induced by an external bias voltage [37]. Such a change in *λ*, albeit tiny, can narrow the potential well felt by each water molecule and, thus, leads to enhanced density oscillations. The opposite case occurs on the outflow side.

**Figure 3.**
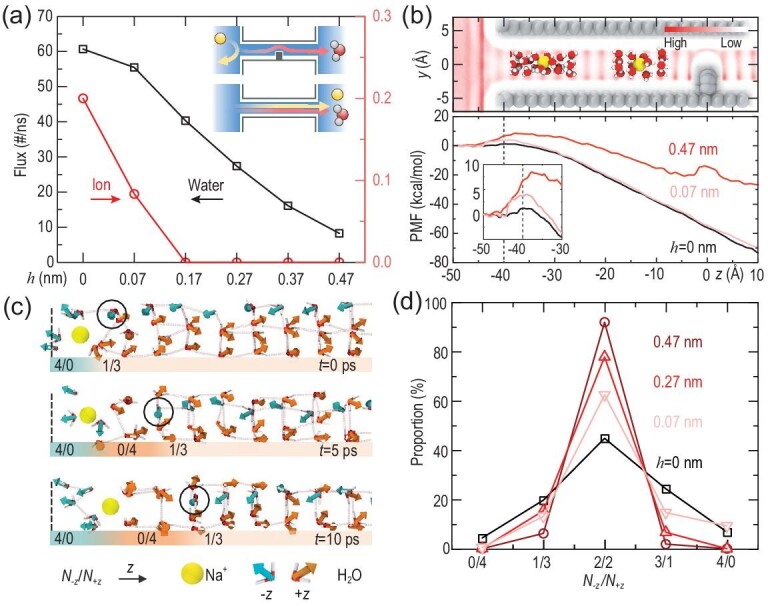
Remote ion blocking due to density oscillations. (a) Net flux of water and ions in the (8,8) Δ-CNTs. Two insets illustrate different transport behaviors of ions in the perturbed and perfect CNTs, respectively. (b) Top panel: density map of water confined in the (8,8) Δ-CNTs with *h* = 0.47 nm. Insets: snapshots of different hydration states of Na^+^ ions at a peak and a valley of the oscillating profile, respectively. Bottom panel: potential of mean force (PMF) of Na^+^ ions in the Δ-CNTs. Insets zoom in to the barriers at the channel entrance whose position is indicated by a dashed line. (c) Gradual evaluation of the water dipole chains from the 1/3 to the 0/4 state as a Na^+^ ion transports along a perfect CNT. *N*_−_*_z_* and *N*_+z_ denote the numbers of water chains with diploes oriented along the *−z* and *+z* directions, respectively. Circles mark the molecules with dipoles flipped by the ion transport. (d) Distribution of *N*_−_*_z_*/*N*_+z_ in perfect and locally perturbated CNTs.

More surprising results arise in flowing ionic water through the channels. Taking salt solution as an example, both Na^+^ and Cl^–^ ions are blocked at the distant entrance, resulting in a zero flux of ions if *h* > 0.07 nm in a (8,8) Δ-CNT, even when the pressure is up to 100 MPa (Fig. 3a, insets). Such a fine control of ion transport by adjusting the perturbation is shown to be general across all the confined systems examined (Fig. S11).

To understand the remote blocking of ions, we check to see if the water is compressed by the baffle inside the Δ-CNTs. Yet, the calculated overall densities of water in the CNTs do not show a meaningful difference from the case of a perfect CNT. This stands in contrast to the gating of nanopores induced by field-induced water compression [38]. Instead, the density map of water molecules shows an oscillating pattern throughout the Δ-CNTs (Fig. 3b, top)—much like quantized energy levels in quantum systems—such that the hopping of ions is required for their transport. Taking Na^+^ ions as an example, they prefer to stay at valleys of *ρ* for a favorable hydration state. Staying at the peaks will disrupt the water structure (Fig. 3b, top insets). This abrupt change of local water structure prohibits the ion passage. Indeed, the calculated potential of mean forces (PMFs) for the ions displays notable barriers near the inflow entrance (Fig. 3b, bottom for Na^+^ ions, Fig. S12 for Cl^–^ ions). These barriers indicate a repulsive force to the ions. The PMF barrier is as high as 9 kcal/mol for a (8,8) Δ-CNT with *h* = 0.47 nm, equivalent to a force of ∼0.5 nN. The PMF barrier still reaches 3 kcal/mol when *h* is as low as 0.07 nm (Fig. 3b).

The PMF barrier can be understood by looking into the hydration status of ions near the entrance. We examined water dipole distributions in CNTs by classifying all molecules into two states according to their projected dipole orientations onto the *z* direction, denoted as −*z* and +*z* states, respectively (Fig. 3c, cyan and orange arrows). The similarly oriented molecules form dipole chains, with *N*_+_*_z_* and *N*_−z_ denoting their numbers. There are four dipole chains inside the (8,8) CNT. These chains evolve among different states including *N*_−_*_z_*/*N*_+z _= 1/3, 2/2 and 3/1. The 2/2 state has the highest probability over time, followed by the 1/3 and 3/1 states (Fig. 3d). An ion entering the channel leads to reorientation of water dipoles nearby, towards achieving a proper hydration structure (Fig. 3c). This reorientation distorts the dipole chains and, hence, increases the potential energy. The ion passage is more difficult in the 2/2 state than in the 1/3 and 3/1 states since one more dipole chain needs to be rearranged in the 2/2 state. The probability of the 2/2 state is significantly raised by the localized perturbation, i.e. the water oscillations (Fig. 3d), thereby resulting in the higher PMF barrier. The probability of the 2/2 state monotonically depends on the baffle height *h* (or the PMF barrier, Fig. S13).

When we consider these findings for practical applications, the benefits are clear. Blocking ions from entering channels using distant disturbance offers great advantages over the conventional means based on steric effect or coulomb interaction. These findings open a promising new path for developing seawater desalination technology that will potentially eliminate the issues of ion scalant and membrane backwashing that have plagued current desalination technologies.

## CONCLUSIONS

Our extensive atomistic simulations combined with model analyses have shown unusual density oscillations in nanochannel-confined water with localized perturbation. The oscillations exist not only in static water but also in flowing states, with a characteristic decay with distance. This profile of water density provides a vivid analogue to the Friedel oscillations that occur around a defect in Fermi liquids. Of practical interest is that the oscillations are independent of specific channel geometries and the physical form of the potential perturbation. Surprisingly, the oscillations can sustain for up to 10 nm, posing a remotely propagating hydrodynamic force that repels ions from the channel entrance. The described systems exhibit the characters of both 1D and 0D confined water, which, thus, can be referred to as fractionally confined water. This work reveals the global impact on ion transport of oft-ignored, localized perturbations in nanochannels, as well as the unexpected complexity in transport behaviors in practical confined systems. It also opens a new prospect for understanding the behaviors of confined water and will stimulate further study of other quantum-like phenomena in water.

## METHODS

All the simulation systems were generated using the VMD software, along with nanotube, solvate, pbctools and autoionize plugins for the modeling process [39]. Each simulation system had two ionic solution reservoirs separated by a channel system. The channel system was composed of two parallel graphene sheets with holes at the center and a CNT vertically aligned in between. For the slit systems, multilayered graphene sheets were used. Carbon atoms in the middle of the sandwiched graphene layers were removed to leave zigzag edges and form 1D channels. In each model, a zigzag-edged graphene fragment was placed perpendicular to the channel axis as a baffle in the middle of the channel. In the verifying simulation that mimicked the experimental set-up of a slit channel under real conditions [14], an extra benzene ring was protruded out of the zigzag edges in the channel. Models without baffles were also used as control groups.

MD simulations were performed using the NAMD2 software package [40]. Parameters of the CHARMM36 force field were used, with carbon atoms modeled as the type CA atoms [41]. The concentration of the NaCl solution was 0.6 mol/L and NBfix parameters were used for ions. The TIP4P water model was used for the production runs [42] and the TIP3P water model was used for verifying the results [43] (Fig. S14). Periodic boundary conditions were applied in all directions. The particle mesh Ewald (PME) method was employed to describe long-range electrostatic interactions [44]. A time step of 2 fs was used with trajectories collected every two picoseconds. The temperature was set to 300 K using Langevin dynamics and a 1-atom pressure was used by applying the Langevin piston method [45]. Each system was initially minimized for 1000 steps, followed by a 1 ns equilibrium using an NVT canonical ensemble (a constant number of particles, volume and temperature) and a 5 ns equilibrium using NPT ensemble (a constant number of particles, pressure and temperature). Production runs lasted for 100 ns and the trajectories from the last 60 ns were used for analysis. The hydrostatic pressure difference was created along the *z* direction by applying a constant force *f* to oxygen atoms in a slab of water away from the channel so as to maintain continuous water flow through the channel [46].

The PMFs for ions were calculated using umbrella sampling [47]. The reaction coordinate *z* was chosen along the channel axis with an interval of 0.5 Å for each window. In each window, a harmonic potential of 5 kcal/mol/Å^2^ was applied on the *z* coordinate of the ion during the sampling process. Simulations were conducted for 1 ns after equilibrium, with the last 800 ps used for data analysis. The obtained histograms were well overlapped and were combined in an unbiased fashion using the weighted histogram analysis method (WHAM) to generate the PMF curves [47,48].

## Supplementary Material

nwab214_Supplemental_File
