# Supplementary material for: An analog of Friedel oscillations in nanoconfined water
Source: Natl Sci Rev. 2021 Nov 29;9(9):nwab214. doi: 10.1093/nsr/nwab214 (PMC9529359; doi:10.1093/nsr/nwab214)
Supplement: nwab214_Supplemental_File [file nwab214_supplemental_file.docx]

Supplementary Material for

**An Analog of Friedel Oscillations in Nanoconfined Water**

Minmin Xue, Zhili Hu, Hu Qiu, Chun Shen, Wanlin Guo^*^ and Zhuhua Zhang^∗^

Key Laboratory for Intelligent Nano Materials and Devices of Ministry of Education, State Key Laboratory of Mechanics and Control of Mechanical Structures, and Institute for Frontier Science, Nanjing University of Aeronautics and Astronautics, Nanjing 210016, China.

*Email: wlguo@nuaa.edu.cn, chuwazhang@nuaa.edu.cn.

**
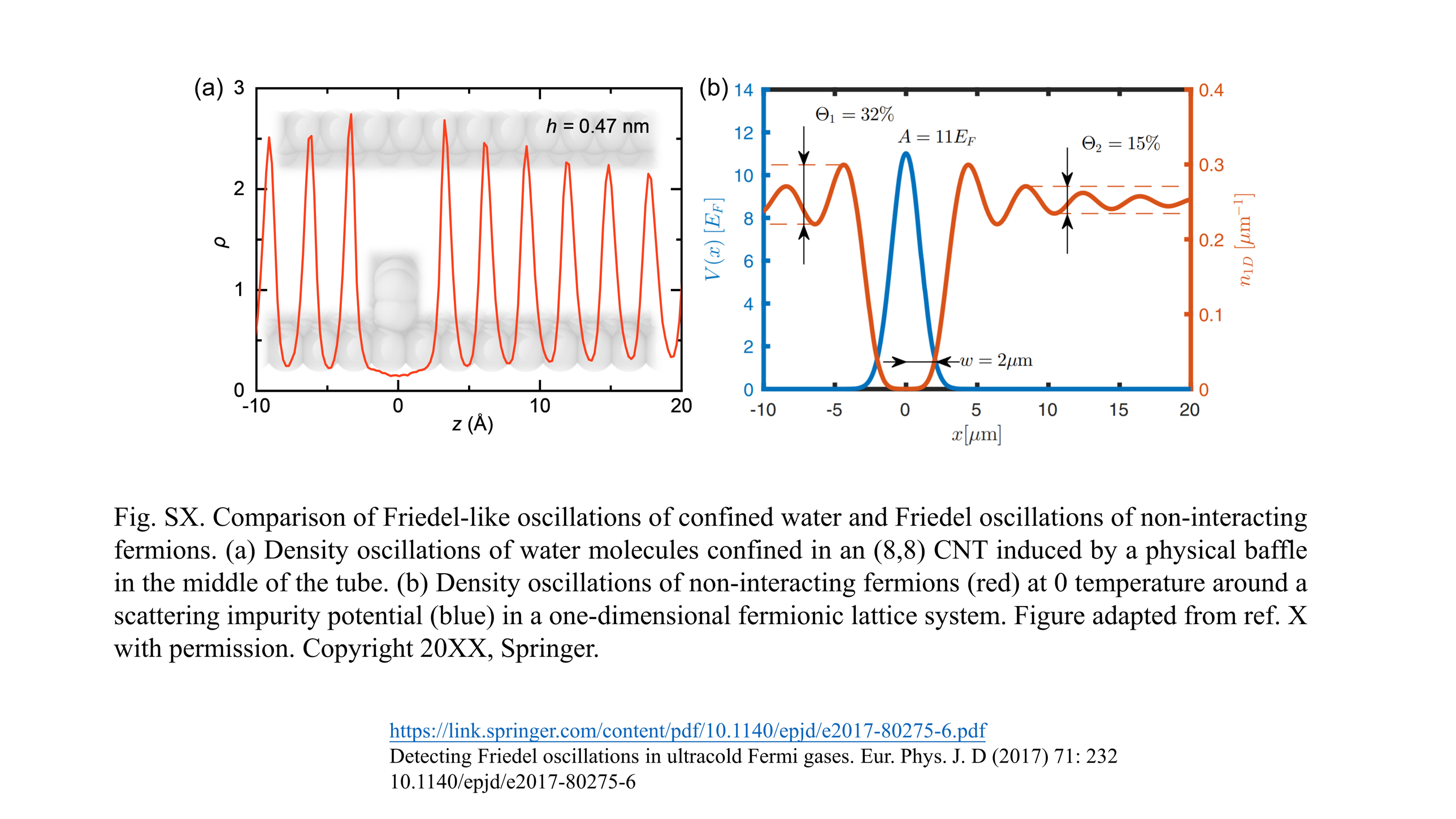
**

**Figure S1.** Comparison of Friedel-like oscillations of confined water and Friedel oscillations of non-interacting fermions. (a) Density oscillations of water molecules confined in an (8,8) Δ-CNT induced by a physical baffle in the middle of the tube. (b) Density oscillations of non-interacting fermions (red) at a temperature of 0 K around a scattering impurity potential (blue) in a one-dimensional fermionic lattice system[1]. Figure adapted from ref. 1.


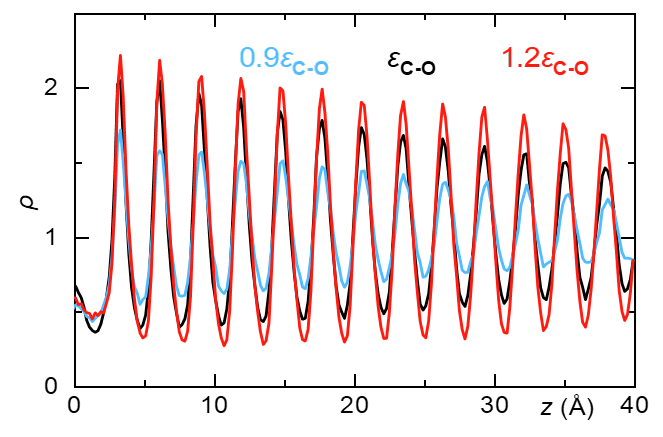


**Figure S2.** Density oscillations in the (8,8) Δ-CNT (*h*=2.7 Å) with different water-CNT interactions. The parameters *ε’*_C-O_ in the modified Leonard-Jones interaction function are set to 0.9 (blue) or 1.2 (red) times the original value *ε*_C-O_.


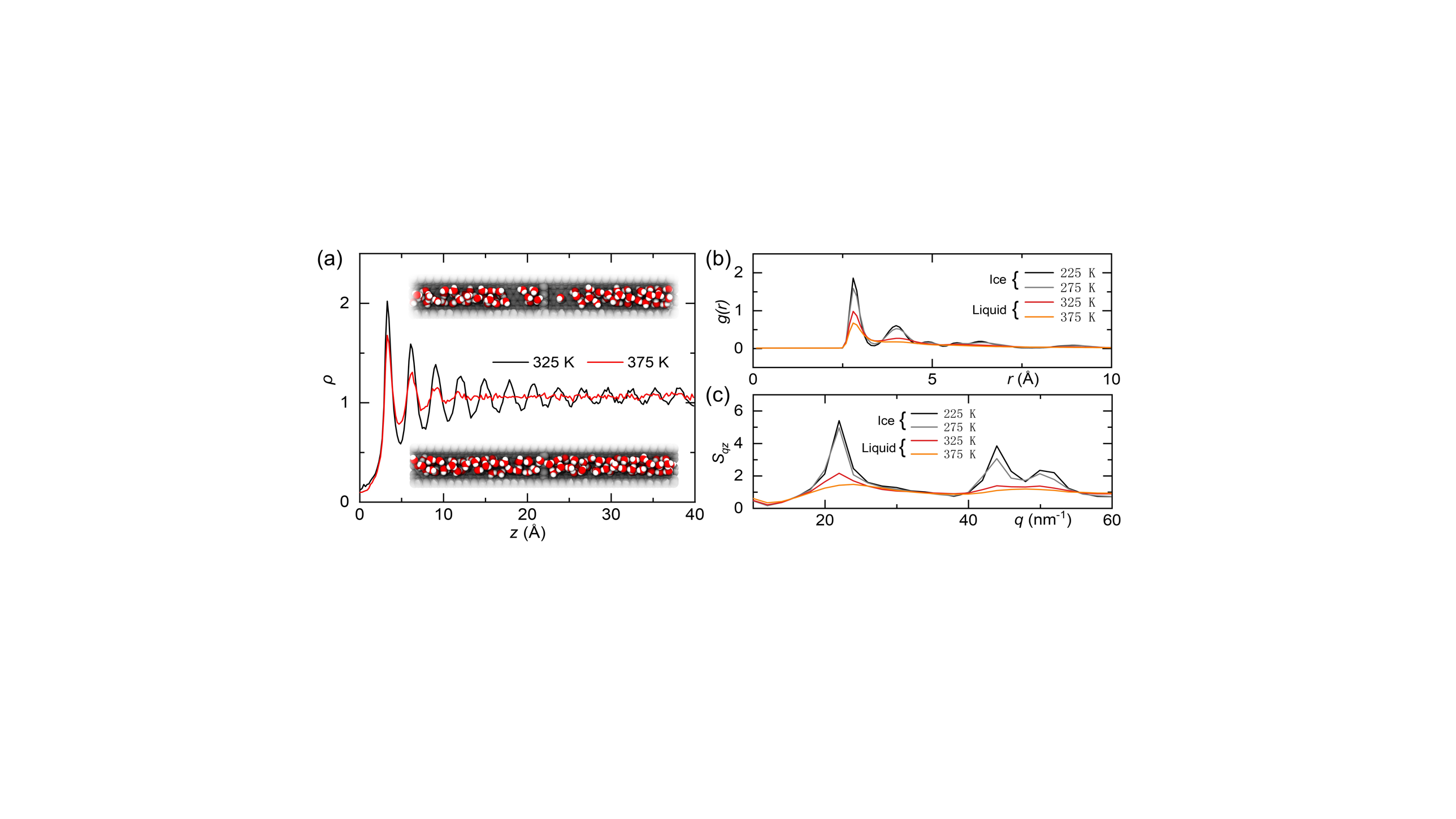


**Figure S3.** Density oscillation of 1D confined water in liquid state with disordered structure. (a) Density oscillation profiles of water confined in the (8,8) Δ-CNT (*h*=4.7 Å) at 325 and 375 K. The top and bottom insets show discrete and disordered water structures at 325 and 375 K, respectively. (b) Calculated radial distribution functions and (c) structure factors of oxygen atoms at different temperatures.

**Figure S4.** Density oscillations in systems of a collapsed CNT and a slit channel with a baffle (external pressure of 100 MPa was applied for the latter case).


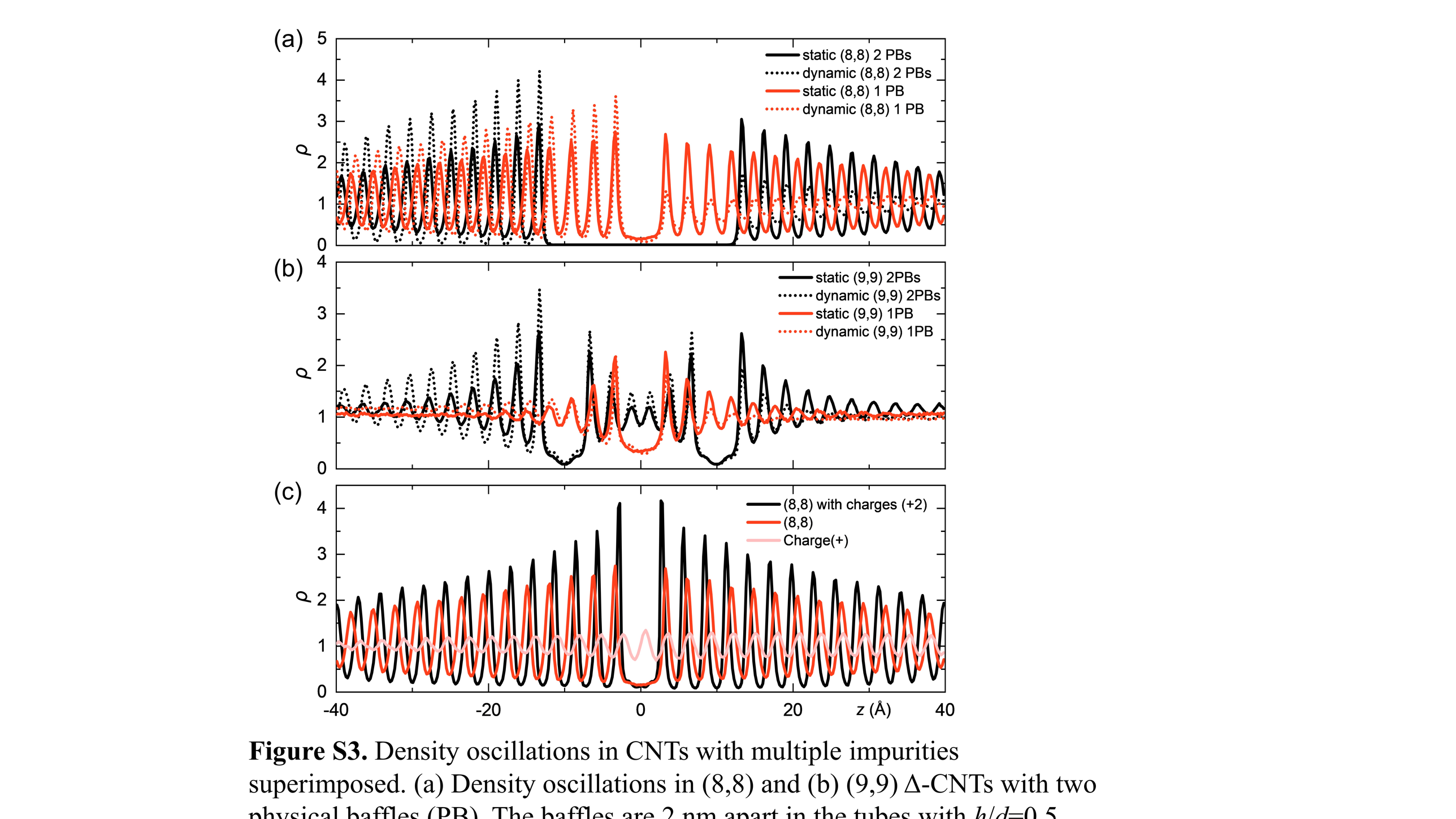


**Figure S5.** Density oscillations in CNTs with multiple impurities superimposed. (a) Density oscillations in (8,8) and (b) (9,9) Δ-CNTs with two physical baffles (PB). The baffles are 2 nm apart in the tubes with *h*/*d*=0.5. Oscillations in dynamic states with an external pressure of 100 MPa were shown for comparison. (c) Density oscillations of water confined in (8,8) Δ-CNTs with one baffle in the middle, with +1 charges imposed on two carbon atoms in the baffle.


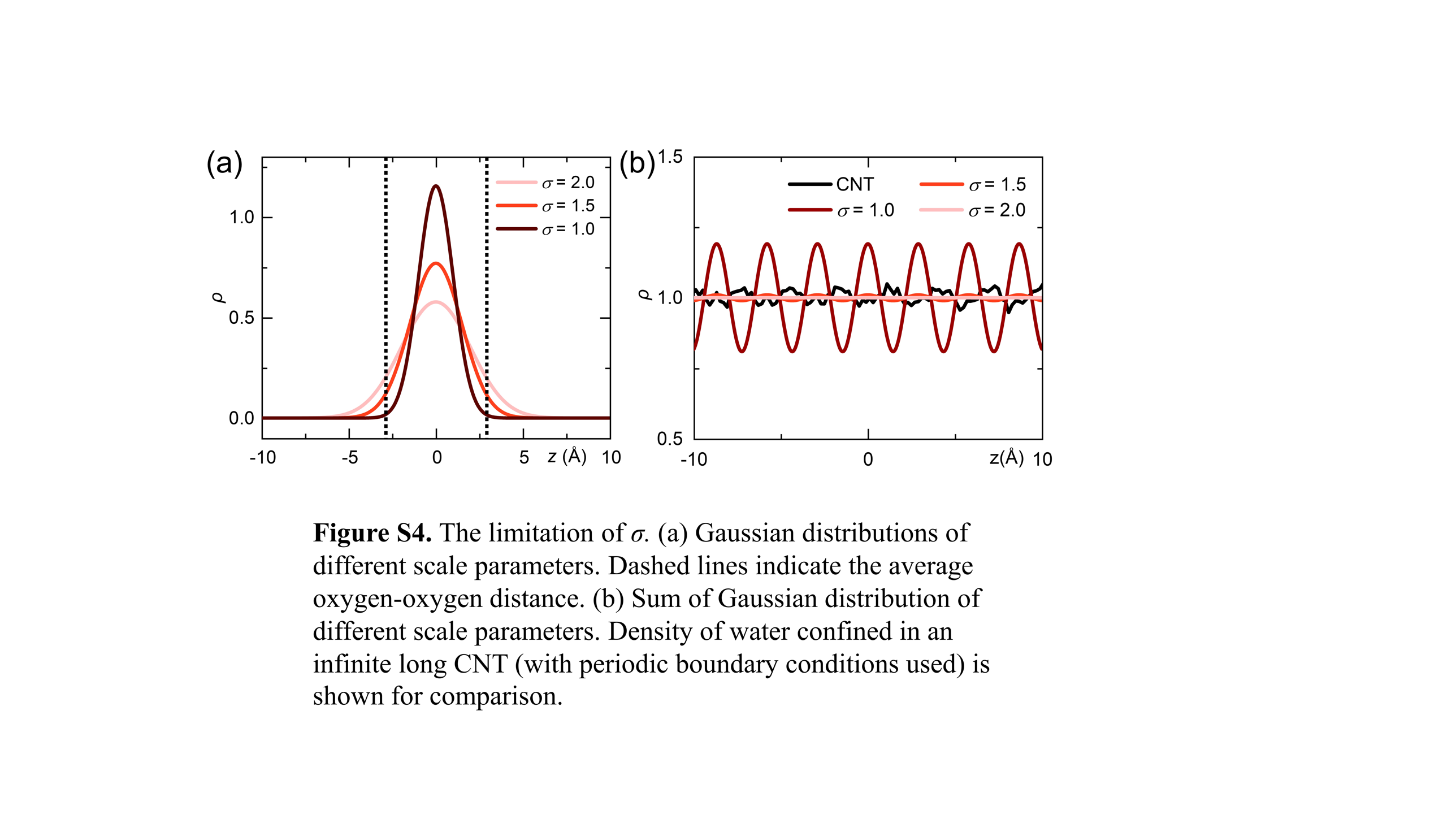


**Figure S6.** The limitation of *σ.* (a) Gaussian distributions of different scale parameters. Dashed lines indicate the average oxygen-oxygen distance. (b) Sum of Gaussian distribution of different scale parameters. Density of water confined in an infinite long CNT (with periodic boundary conditions used) is shown for comparison.


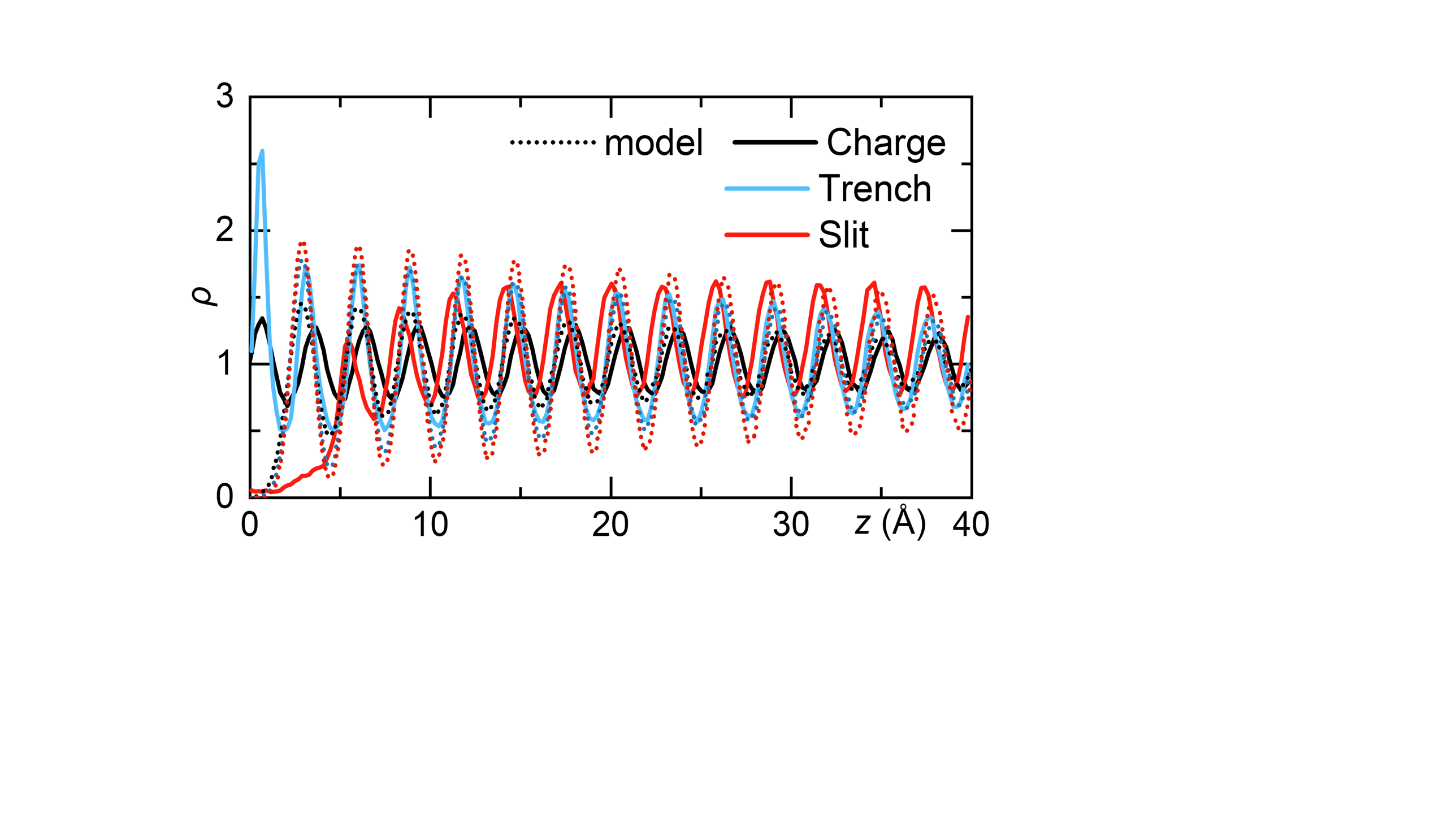


**Figure S7.** Analytical model describing systems with other forms of perturbations. *g* and *σ* in equation 3 are chosen as 0.97 and 0.8, 0.97 and 0.65, and 0.98 and 0.65 for perturbations of charge impurity, trench and slit, respectively.


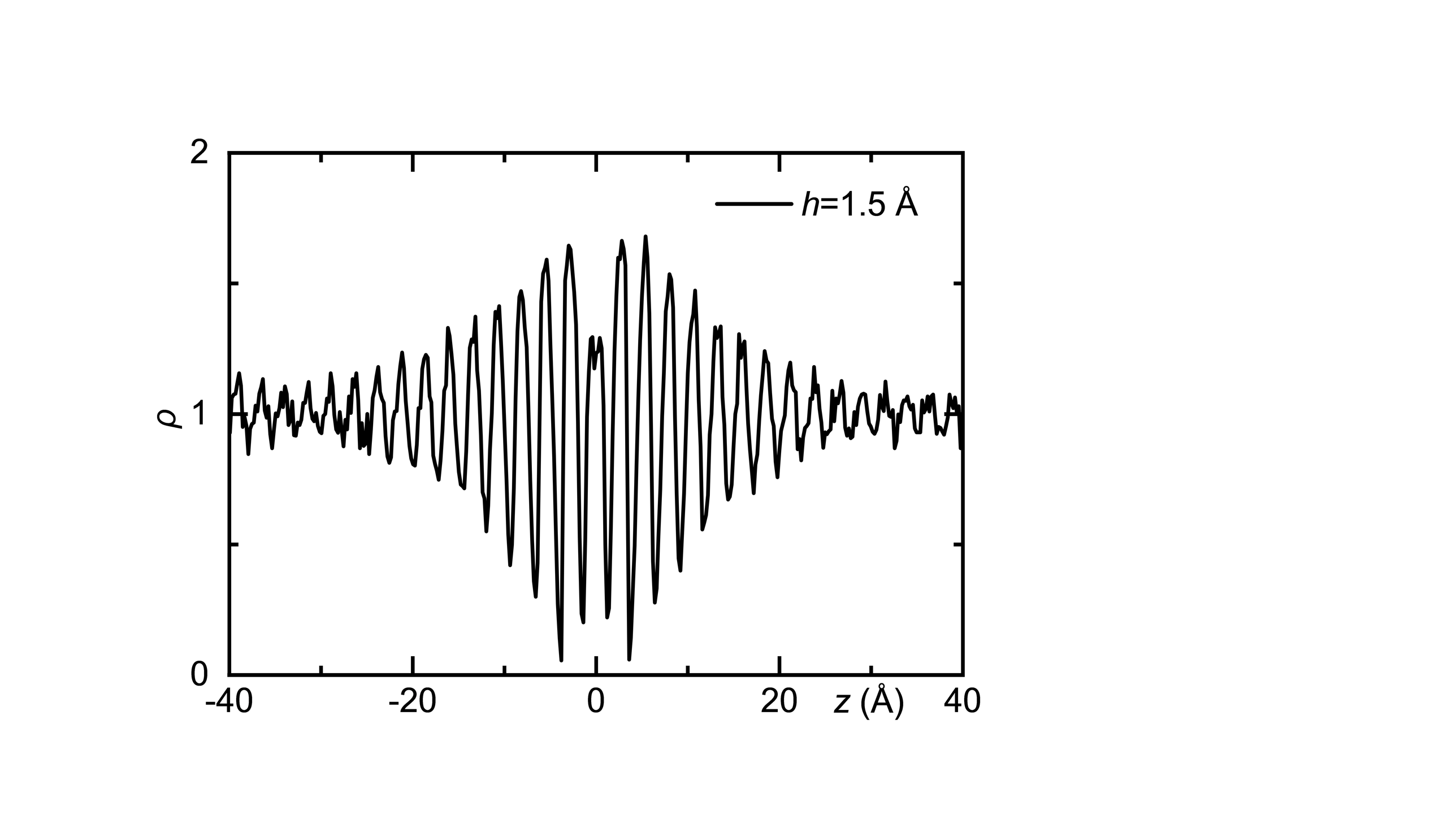


**Figure S8.** Calculated density oscillations in a (6,6) Δ-CNT with a baffle height of *h*=1.5 Å.

**Figure S9.** Linear relationship between *g* and <cos*θ>* in Δ-CNTs. Inset shows the angle *θ* between dipole vectors of adjacent water molecules along *z* axis.

**Figure S10.** Friedel-like density oscillations of the confined water flowing in (8,8) Δ-CNTs under an external pressure of 100 MPa.


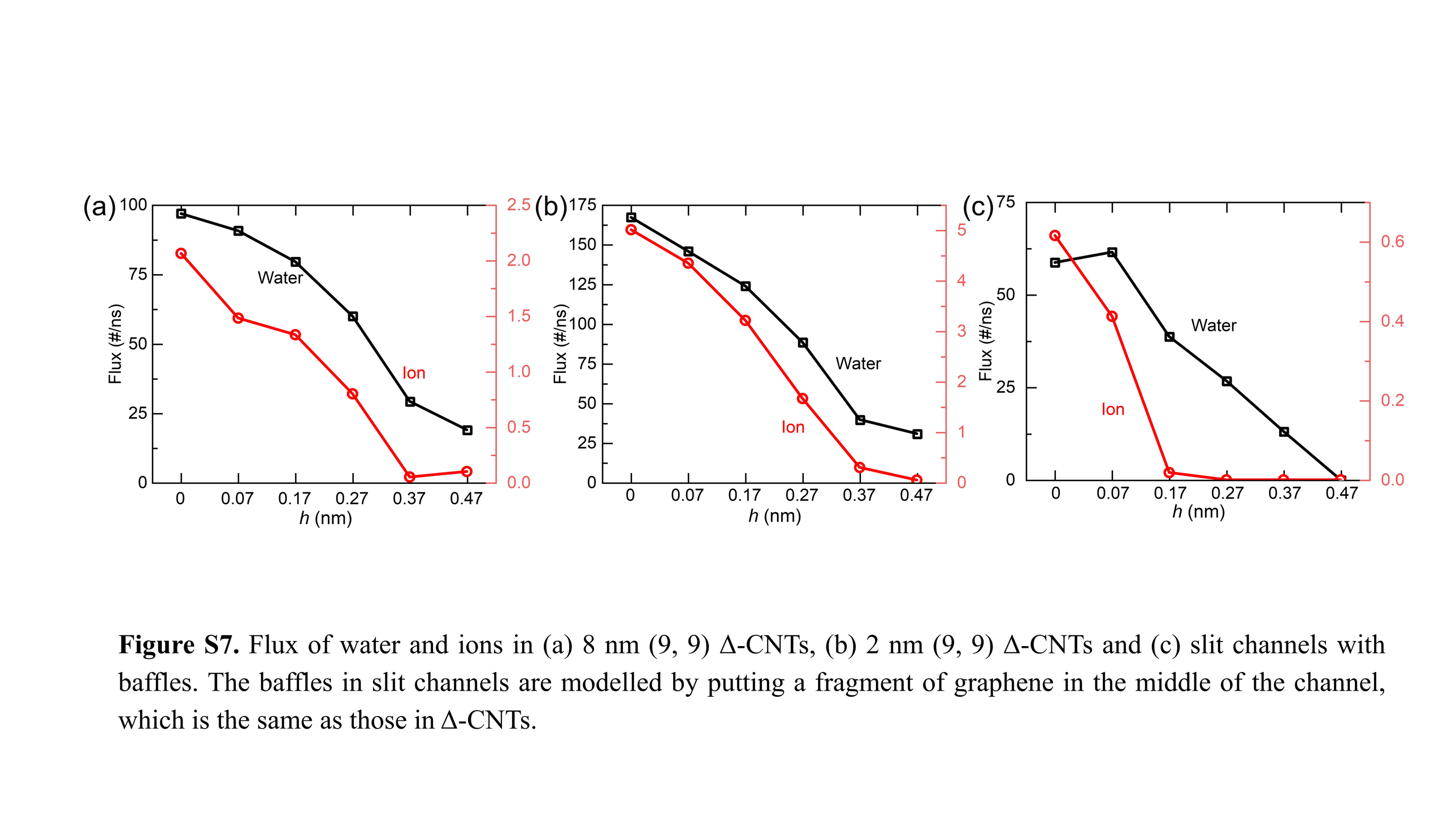


**Figure S11.** Flux of water and ions in (a) 8 nm (9, 9) Δ-CNTs, (b) 2 nm (9, 9) Δ-CNTs and (c) slit channels with baffles. The baffles in slit channels are modelled by putting a fragment of graphene in the middle of the channel, which is the same as those in Δ-CNTs.


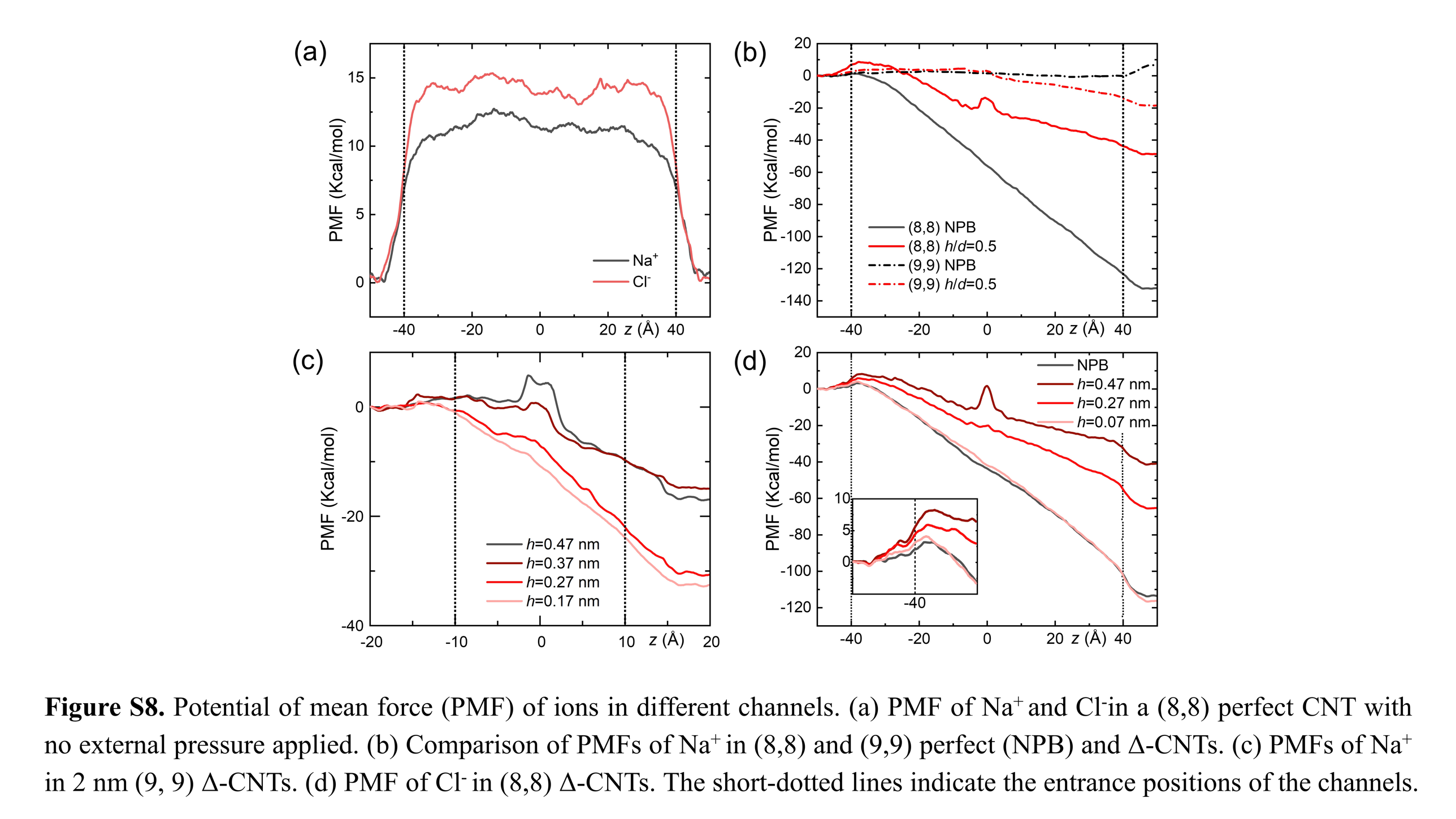


**Figure S12.** Potential of mean force (PMF) of ions in different channels. (a) PMF of Na^+^ and Cl^-^in a (8,8) perfect CNT with no external pressure applied. (b) Comparison of PMFs of Na^+^ in (8,8) and (9,9) perfect (NPB) and Δ-CNTs. (c) PMFs of Na^+^ in 2 nm (9, 9) Δ-CNTs. (d) PMF of Cl^-^ in (8,8) Δ-CNTs. The short-dotted lines indicate the entrance positions of the channels.

**Figure S13.** Δ*E* as a function of *h* in (8,8) Δ-CNTs. Δ*E* is defined as the discrepancy of energy barriers between Δ-CNTs and the perfect (8,8) (NPB) CNT at the entrance position. The dotted line indicates the energy barrier for the perfect (8,8) CNT.


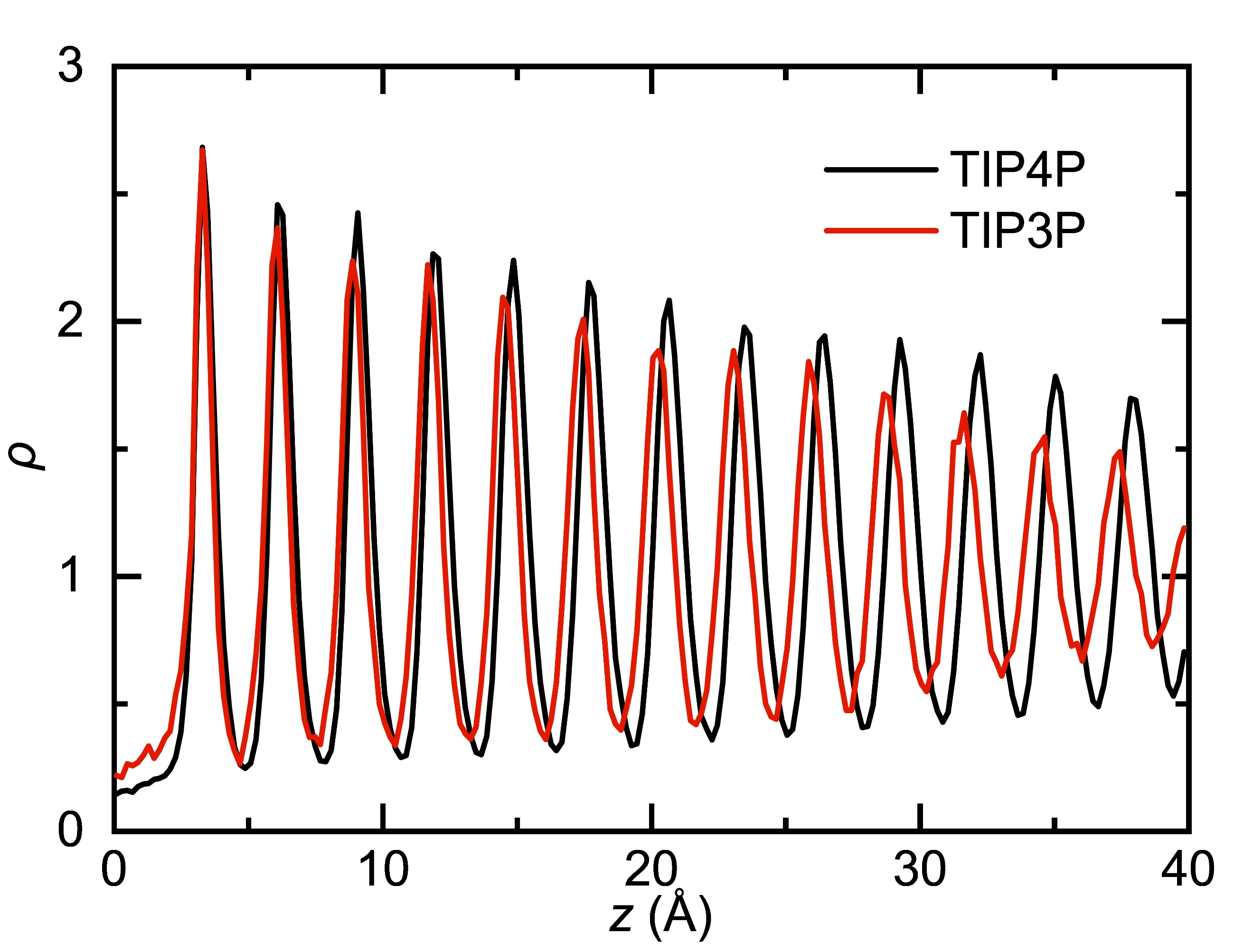


**Figure S14.** Density oscillations obtained using TIP3P and TIP4P water models in the (8,8) Δ-CNT with a baffle height of *h*=4.7 Å at the same temperature level of 80 K above the corresponding melting points.

**References**

1. Riechers K, Hueck K, Luick N *et al.* Detecting Friedel oscillations in ultracold Fermi gases. *Eur Phys J D* 2017; **71**: 232.
